# Neuroprotective Effects of OMO within the Hippocampus and Cortex in a D-Galactose and A*β*_25–35_-Induced Rat Model of Alzheimer's Disease

**DOI:** 10.1155/2020/1067541

**Published:** 2020-10-10

**Authors:** Shaodong Deng, Hongmei Lu, Honggang Chi, Ying Wang, Xiao Li, Haiyi Ye

**Affiliations:** ^1^Scientific Research Platform, The Second Clinical Medical College, Guangdong Medical University, Dongguan 523808, China; ^2^Department of Traditional Chinese Medicine, The Second Clinical Medical College, Guangdong Medical University, Dongguan 523808, China; ^3^Department of Emergency, Nanfang Hospital, Southern Medical University, Guangzhou 510515, China

## Abstract

*Morinda officinalis* F.C. How. (Rubiaceae) is a herbal medicine. It has been recorded that its oligosaccharides have neuroprotective properties. In order to understand the oligosaccharides extracted from *Morinda officinalis* (OMO), a systematic study was conducted to provide evidence that supports its use in neuroprotective therapies for Alzheimer's disease (AD). AD rat models were prepared with D-galactose and A*β*_25–35_. The following groups were used in the present experiment: normal control group, sham-operated group, model group, Aricept group, OMO low-dose group, OMO medium-dose group, and OMO high-dose group. The effects on behavioral tests, antioxidant levels, energy metabolism, neurotransmitter levels, and AD-related proteins were detected with corresponding methodologies. AD rats administered with different doses of OMO all exhibited a significant (*P* < 0.05) decrease in latency and an increase (*P* < 0.05) in the ratio of swimming distance to total distance in a dose-dependent manner in the Morris water maze. There was a significant (*P* < 0.05) increase in antioxidant enzyme activities (SOD, GSH-Px, and CAT), neurotransmitter levels (acetylcholine, *γ*-GABA, and NE and DA), energy metabolism (Na^+^/K^+^-ATPase), and relative synaptophysin (SYP) expression levels in AD rats administered with OMO. Furthermore, there was a significant (*P* < 0.05) decrease in MDA levels and relative expression levels of APP, tau, and caspase-3 in AD rats with OMO. The present research suggests that OMO protects against D-galactose and A*β*_25–35_-induced neurodegeneration, which may provide a novel strategy for improving AD in clinic.

## 1. Introduction

*Morinda officinalis* F.C. How. (Rubiaceae) is a traditional Chinese medicine that has good kidney-invigorating and spermatogenic effects. This medicine is at the forefront of the clinical application of kidney-tonifying Chinese medicines. According to traditional theories of Chinese medicine, cerebral diseases can be prevented by invigorating the kidneys. It has been proven that carbohydrates account for nearly 50% of the content of dried radix morindae, and that these are mainly oligosaccharides [1]. A recent study revealed that oligosaccharides extracted from *Morinda officinalis* (OMO) can significantly enhance the learning and memory abilities of rats with dementia, and that these can improve dementia symptoms [2]. Specifically, oligosaccharide monomers, such as Bajijiasu and nystose, exhibit various biological activities, such as neuroprotective [3], antidementia [4], antiosteoporosis [5], and antidepressant [6] effects, suggesting that OMO is a good choice for screening potential drugs.

Alzheimer's disease (AD), which is also called senile dementia, is the most common chronic neurodegenerative disorder, which severely impacts quality of life of aged individuals. According to one report, there were approximately 50 million AD patients worldwide in 2018 [7]. This number is predicted to increase to 153 million, three times of the present incidence, by 2050. Furthermore, it was estimated that the global cost of AD was approximately 1,000 billion dollars in 2018, which could increase to 2,000 billion dollars by 2030. Therefore, the effective prevention and treatment of AD are critical topics worldwide. AD is caused by various factors but is commonly characterized by neurofibrillary tangles (NFT), senile plaques (SP), and loss of numerous neurons. At present, its pathogenesis mainly includes *β*-amyloid protein theory, tau protein theory, hypofunction of the cholinergic system, and glutamic acid transmitter disorder. With the advances in research, various theories have been proposed, such as cell cycle regulatory protein abnormalities, oxidative stress, inflammatory mechanisms, mitochondrial function disorders, and autophagy [8–11]. Numerous studies have already investigated the mechanism of AD. However, no effective methods or drugs are available to prevent or slow the progression of AD.

**Figure 1:**
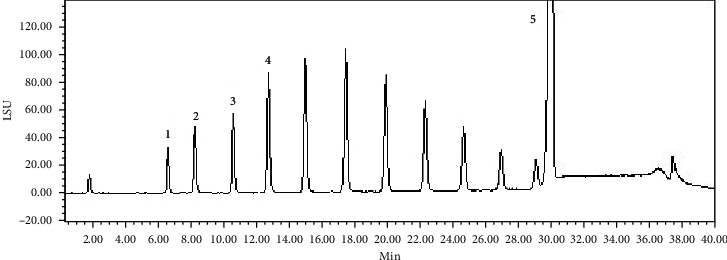
The HPLC-ELSD results of oligosaccharides extracted from *Morinda officinalis*. (1) Sucrose, (2) kestose, (3) nystose, (4) 1F-Fructofuranosyl nystose, and (5) Bajijiasu.

Based on previous research studies on Bajijiasu [12, 13], the investigators proposed that OMO may have a potential to treat AD. OMO were extracted with over 98% purity, and the HPLC-ELSD analysis [14] revealed that OMO contained 5.48% sucrose, 7.25% kestose, 8.89% nystose, 10.57% 1F-Fructofuranosyl nystose, and 29.37% Bajijiasu (Figure 1). Considering the pathological symptoms of AD, an AD model was established with d-galactose and A*β*_25–35_. Pharmacological, analytical chemistry, and molecular biological analyses of traditional Chinese medicine were performed, and the Morris water maze, Coulomb array electrochemistry, and enzyme labeling methods were used to investigate the behavior, neurotransmitter levels, energy metabolism, oxidative stress, and cholinergic energy. The expression levels of beta-APP, tau, caspase-3, and SYP were detected by Western blot on hippocampal tissues obtained from AD model rats. Overall, the present study provides a scientific basis for research on the use of Chinese medicine for the prevention and treatment of AD.

## 2. Materials and Methods

### 2.1. Drugs and Reagents

A*β*_25–35_ (A4559) and d-galactose (G0625) were purchased from Sigma-Aldrich (USA). Aricept was purchased from Eisai Pharmaceutical Co., Ltd. (China). The detection kits for superoxide dismutase (SOD), malondialdehyde (MDA), catalase (CAT), Na^+^/K^+^-ATPase, acetylcholine (ACh), acetylcholinesterase (AChE), nitric oxide (NO), and glutamate (Glu) were all purchased from Nanjing Jiancheng Bioengineering Institute (China). The level of *γ*-aminobutyric acid (GABA) was measured using a kit purchased from Bogoo Biotechnology Co., Ltd. The following primary antibodies were used for the present study: anti-APP (Cell Signaling, Cat. #2452), anti-SYP (Cell Signaling, Cat. #5461), anti-caspase-3 (Cell Signaling, Cat. #9665), anti-GAPDH (Cell Signaling, Cat. #5174), and anti-p-tau ser404 (Epitomics, Cat. #2691-1).

### 2.2. Experimental Animals

Six-month-old male SD rats (300–350 g) were purchased from the Experimental Animal Center at Guangzhou University of Chinese Medicine. These rats were housed in rooms with controlled 12 hour light/dark cycles, temperature, and humidity and were given access to food and water ad libitum. According to a literature report [15], all SD rats were allowed to adapt to the environment for one week and were trained in the Morris water maze until a stable performance was achieved over two days. All animal experiments were performed with the approval of the Animal Ethics Committee of Guangdong Medical University.

### 2.3. Animal Grouping

The animals were divided into seven groups (*n* = 16) as follows: normal control group, rats with no treatment; sham-operated group, non-AD control rats that received distilled water; model group, AD rats induced with d-galactose and A*β*_25–35_, but without treatment; Aricept group, AD rats administered with Aricept of 0.4 mg/kg per day orally; low-dose OMO group (120 mg/kg per day), middle-dose OMO group (240 mg/kg per day), and high-dose OMO group (480 mg/kg per day), all orally. Each group was treated for four weeks. The dosage of OMO was converted from the daily dosage in Chinese Pharmacopoeia about *Morinda officinalis* (Rubiaceae).

### 2.4. Alzheimer's Disease Induction

The experimental groups and sham-operated group were intraperitoneally injected daily with 150 mg/kg of d-galactose, according to a literature [16, 17], or saline, respectively, for seven consecutive weeks. In the fifth week in a row, rats were anesthetized with 45 mg/kg of pentobarbital and placed in a stereotaxic frame. The injections were carried out bilaterally above the hippocampal CA1 area at the following stereotactic coordinates: anterior-posterior (AP), −3.6 mm from the bregma; mediolateral (ML), ±2.5 mm from the midline; and dorsoventral (DV), 3.0 mm below the surface of the skull. The A*β*_25–35_ was adjusted to 5 g/L in saline in a 37°C incubator for seven days before the injection. A total of 2 *μ*L of A*β*_25–35_ solution or saline was prepared for each injection site at a rate of 0.4 *μ*L/min. After the injection, the needle remained in position for 10 minutes before it was slowly withdrawn. The animals were injected intramuscularly with penicillin and closely monitored for signs of pain before recovery.

### 2.5. Behavioral Tests

The Morris water maze was used to test the learning, memory, and cognitive abilities of rats, as previously described [18]. The time taken by rats to find the platform (escape latency) within 120 seconds was recorded. The place navigation experiment was performed twice a day for four consecutive days. The ratio of swimming distance in the target quadrant to total swimming distance and the number of times each rat crossed the original platform location in 90 seconds was measured. The spatial probe test was performed on day one.

### 2.6. Collection of Brain Tissue

After the final behavioral test, all rats were sacrificed after fasting for 12 hours, and the whole brain was rapidly removed. Then, the hippocampus and cortex of these rats were rapidly dissected and placed into freezer tubes. Then, the tissue was stored in a freezer at −80°C.

### 2.7. Enzymatic Assays

The hippocampus and cortex of each rat were homogenized in nine volumes of cold saline on ice and centrifuged at 3,500 g for 10 minutes at 4°C. Then, the supernatants were collected for testing. The activities of SOD, GSH-Px, CAT, AChE, NOS, and Na^+^K^+^-ATPase and the MDA, Ach, Glu, GABA, and NO levels were measured using commercial detection kits (Nanjing Jiancheng Bioengineering Institute, Nanjing City, China).

### 2.8. Neurotransmitter Assay

The levels of neurotransmitters, including levodopa (L-DOPA), norepinephrine (NE), epinephrine (E), 3, 4-dihydroxyphenylacelic acid (DOPAC), dopamine (DA), 5-hydroxyindole-3-acetic acid (5-HIAA), homovanillic acid (HVA), and 5-hydroxytryptamine (5-HT), in the hippocampus and cortex were measured by HPLC-ECD: Coulomb array electrochemical high-performance liquid chromatography (ESA company, model 5600a-16 channel detector, model 582 solvent delivery system, model 542 automatic injector, Coularray win workstation); chromatographic column: Thermo Syncronis C_18_ (150 × 4.6 mm, 5 *μ*m); and precolumn: Thermo Hypersil ODS (10.0 × 4.0 mm, 2.5 *μ*m). Mobile phase A (0.13 mol · L^−1^ NaH_2_PO_4_, 2.1 mmol · L^−1^ C_8_H_19_NaO_4_S, 0.1 mmol·L^1^ EDTA-2Na, and pH 3.5) : B (methanol) = 83 : 17; current speed 1 mL·min^−1^; column temperature 35°C; automatic injector temperature 4°C; detection voltage 350 mv; and injection volume 15 *μ*L. The 3, 4-dihydroxybenzylamine hydrobromide (DHBA) was used as the internal standard. Finally, the HPLC-ECD method was validated, according to a previous report [19].

### 2.9. Western Blotting Assay

The hippocampus was homogenized in 10 volumes of lysis buffer on ice and centrifuged at 6036 g for five minutes at 4°C. The supernatants were collected, and the protein concentration was determined using a BCA kit. The lysates were separated using SDS-PAGE and transferred onto a polyvinylidene difluoride membrane. Then, the membrane was blocked in 5% defatted milk and probed with primary antibodies on a shaker at 4°C overnight. After washing, the membrane was incubated with HRP-conjugated goat antirabbit and visualized using enhanced chemiluminescence (ECL) reagents.

### 2.10. Statistical Analysis

The results were presented as mean ± SD. The statistical analysis between multiple groups was performed using ANOVA, followed by Tukey HSD tests. *P* < 0.05 was considered statistically significant.

## 3. Results

### 3.1. Effects of OMO on Behavioral Tests

#### 3.1.1. Effects of OMO on Place Navigation

**Figure 2:**
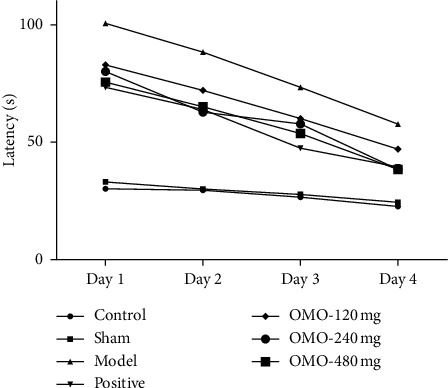
Effect of OMO on latency in D-galactose and A*β*_25–35_-treated AD rats. Control, control group; Sham, sham-operated group; Model, model group (D-galactose and A*β*_25–35_ 10 *μ*g + saline); Positive, positive group (D-galactose and A*β*_25–35_ 10 *μ*g + Aricept 0.4 mg/kg); OMO-120 mg, low-dose OMO group (D-galactose and A*β*_25–35_ 10 *μ*g + OMO 120 mg/kg); OMO-240 mg, middle-dose OMO group (D-galactose and A*β*_25–35_ 10 *μ*g + OMO 240 mg/kg); and OMO-480 mg, high-dose OMO group (D-galactose and A*β*_25–35_ 10 *μ*g + OMO 480 mg/kg). These values given are the mean ± SD. *n* = 10.

The results of the Morris water maze are shown in Figure 2. The latency of each group decreased with the increase in training days. Compared with the normal control group, the latency of the model group was significantly prolonged (*P* < 0.01), while there was no significant difference in the sham-operated group (*P* < 0.05). It was suggested that the d-galactose and A*β*_25–35_-induced AD rat model can induce a decline in the learning and memory abilities of rats, while the operation itself does not cause injury. Compared with that in the model group, the latency in OMO-treated groups was shortened. The difference between the model group and groups administered with middle- and high-doses of OMO was significant (*P* < 0.05). These data show that OMO can ameliorate the d-galactose and A*β*_25–35_-induced memory dysfunction in rats.

#### 3.1.2. Effects of OMO on the Spatial Probe Test

**Figure 3:**
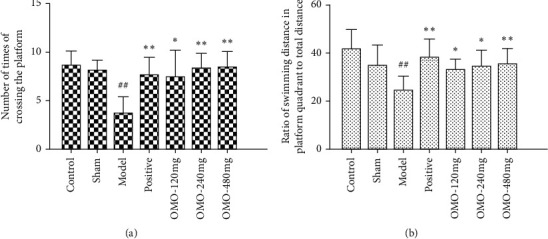
Effect of OMO on the spatial probe test in D-galactose and A*β*_25–35_-treated AD rats. These values are expressed as mean ± SD, *n* = 10. ^##^*P* < 0.01, compared with the normal control group ^*∗*^*P* < 0.05 and ^*∗∗*^*P* < 0.01, compared with the model group.

As shown in Figure 3 and compared with the normal control group, the ratio of swimming distance in the quadrant with the platform to the total distance and the number of times the rat crossed the platform location in the model group significantly decreased (*P* < 0.01). There were also significant differences (*P* < 0.05) between the model group and OMO-treated groups in terms of the ratio of swimming distance in the platform quadrant to total distance and the number of times the rat crossed the platform location. At the same time, the ratio of swimming distance in the platform quadrant to total distance and the number of times the rat crossed the platform location increased with the increase in OMO dosage. These results demonstrate that OMO can ameliorate d-galactose and A*β*_25–35_-induced learning and memory dysfunction in rats.

### 3.2. Neuroprotective Effects of OMO in the D-Galactose and A*β*_25–35_-Induced Rat Model of AD

#### 3.2.1. Effects of OMO on Oxidative Stress Markers in the Hippocampus and Cerebral Cortex in AD Rats

**Figure 4:**
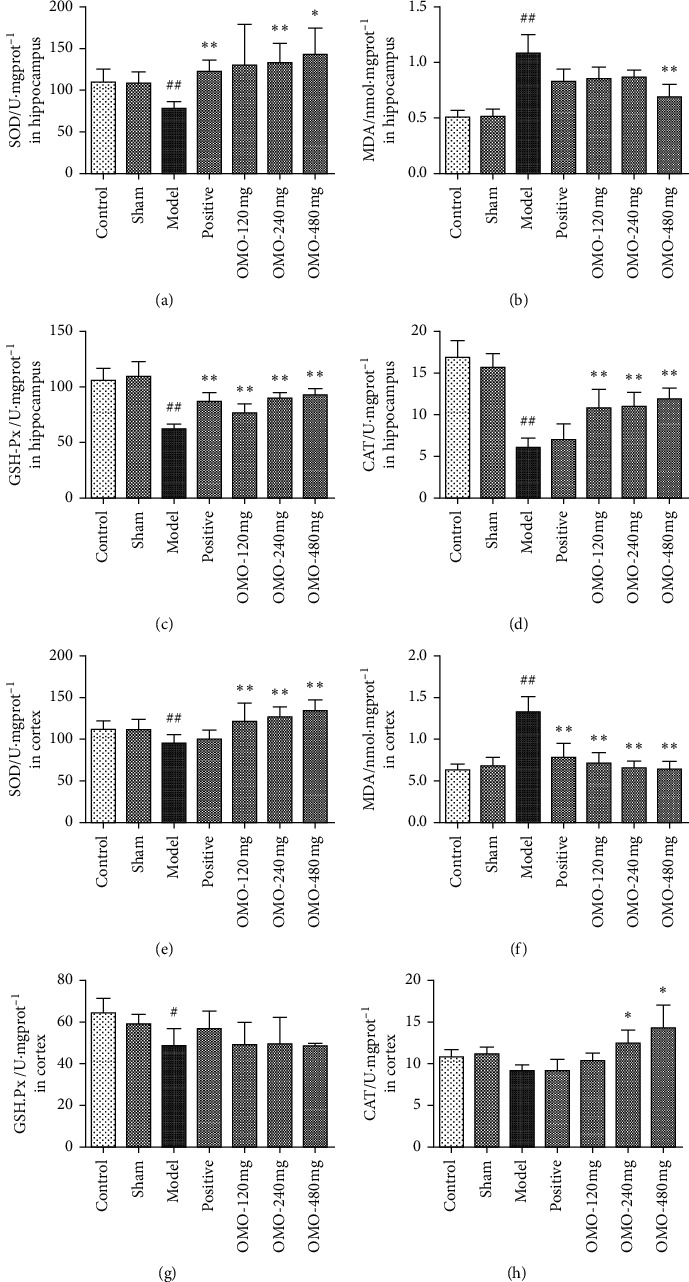
Effect of OMO on oxidative stress markers in the hippocampus and cerebral cortex of D-galactose and A*β*_25–35_-treated AD rats. The graphs show the levels of (a), (e) SOD; (b), (f) MDA; (c), (g) GSH-Px; and (d), (h) CAT in the hippocampus and cortex, respectively. These values are expressed as mean ± SD, *n* = 8. #*P* < 0.05 and ^##^*P* < 0.01, compared with the normal control group. ^*∗*^*P* < 0.05 and ^*∗∗*^*P* < 0.01, compared with the model group.

The activities of SOD, CAT, GSH-Px, and MDA were used as oxidative biomarkers in the D-galactose and A*β*_25–35_-induced AD rat model. As shown in Figure 4, the activities of SOD, CAT, and GSH-Px in the hippocampus and cortex of the model group were lower than those in the normal control group, while the content of MDA was higher than that in the normal control group. Except for the activity of CAT in the cortex, there were significant differences between the overall normal control group and model group (*P* < 0.05). These results suggest that the d-galactose and A*β*_25–35_-induced AD model can decrease the activity of antioxidant enzymes in rat brains. After OMO treatment, the oxidative damage in the brain in each dose group was alleviated to a certain extent. The high-dose OMO treatment significantly increased the activities of SOD, CAT, and GSH-Px in the hippocampus of AD model rats and decreased the content of MDA (*P* < 0.05). Overall, the OMO-treated groups exhibited significant increases in SOD activity in the cortex of AD model rats and decreases in MDA content (*P* < 0.05). These results suggest that OMO can alleviate oxidative damage, and thereby protect brain tissues from oxidative damage.

#### 3.2.2. Effects of OMO on Cholinergic Neurotransmitter Concentrations in the Hippocampus and Cerebral Cortex in AD Rats

**Figure 5:**
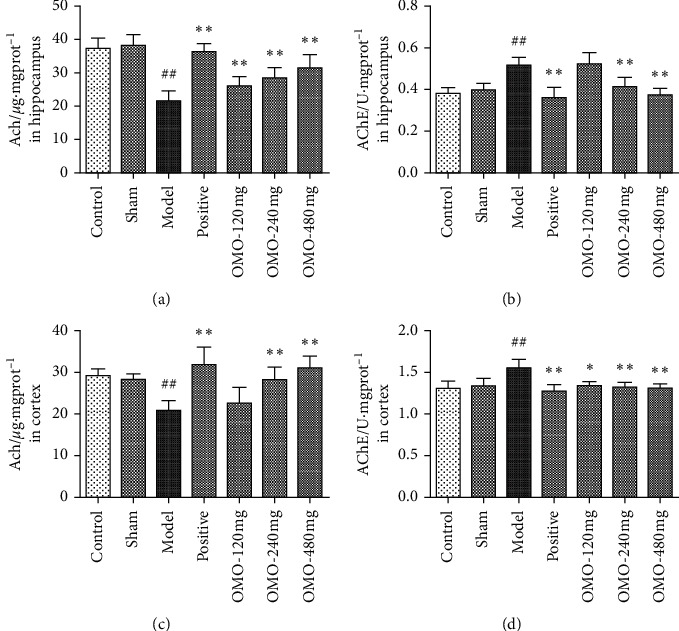
Effect of OMO on cholinergic neurotransmitter concentrations in the hippocampus and cerebral cortex of D-galactose and A*β*_25–35_-treated AD rats. The graphs show the levels of (a), (c) Ach and (b), (d) AchE in the hippocampus and cortex, respectively. These values are expressed as mean ± SD, *n* = 8. ^##^*P* < 0.01, compared with the normal control group. ^*∗*^*P* < 0.05 and ^*∗∗*^*P* < 0.01, compared with the model group.

As shown in Figure 5, the activity of acetylcholinesterase (AChE) in the hippocampus and cortex of the model group was significantly higher than that in the control group (*P* < 0.01), while the level of acetylcholine (Ach) was significantly lower than that in the control group (*P* < 0.01). This suggests that the damage to the cholinergic system observed in d-galactose and A*β*_25–35_-induced AD model rats was similar to that observed in Alzheimer's disease. Compared with the model group, the middle-dose OMO and high-dose OMO groups exhibited significant decreases in the activity of AChE in the hippocampus and cortex and significant increases in the levels of Ach (both, *P* < 0.01). This result suggests that OMO has protective effects on the cholinergic system of AD model rats.

#### 3.2.3. Effects of OMO on Amino Acid Neurotransmitters in the Hippocampus and Cerebral Cortex in AD Rats

**Figure 6:**
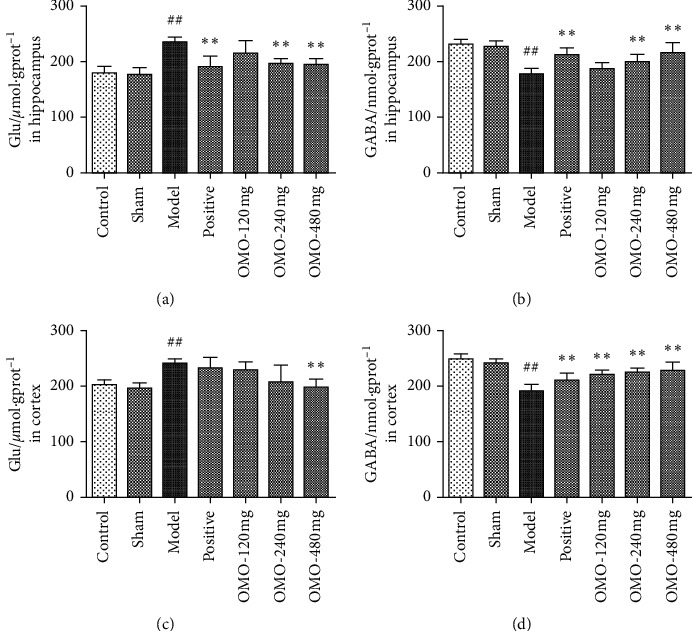
Effect of OMO on amino acid neurotransmitters in the hippocampus and cerebral cortex of D-galactose and A*β*_25–35_-treated AD rats. The graphs show the levels of (a), (c) Glu and (b), (d) GABA in the hippocampus and cortex, respectively. These values are expressed as mean ± SD, (*n*) = 8. ^##^*P* < 0.01, compared with the normal control group; ^*∗∗*^*P* < 0.01 compared with the model group.

As shown in Figure 6, the level of glutamate (Glu) increased, while the level of *γ*-aminobutyric acid (*γ*-GABA) acid decreased in the hippocampus and cortex of rats in the model group, suggesting that the d-galactose and A*β*_25–35_-induced AD model could induce excitatory neurotoxicity. The level of Glu decreased and *γ*-GABA increased after OMO treatment. The differences were significant between the model group and high-dose OMO group (*P* < 0.01).

#### 3.2.4. Effects of OMO on NO/NOS Levels in the Hippocampus and Cerebral Cortex in AD Rats

**Figure 7:**
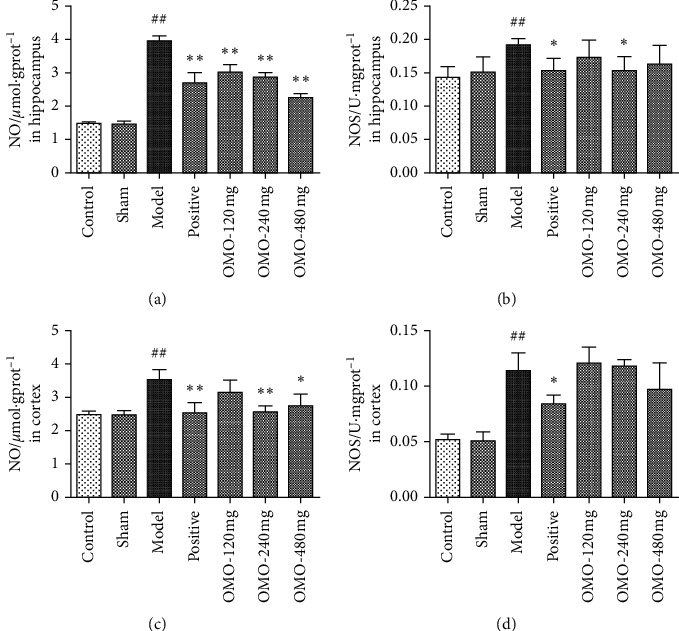
Effect of OMO on NO/NOS levels in the hippocampus and cerebral cortex of D-galactose and A*β*_25–35_-treated AD rats. The graphs show the levels of (a), (c) NO and (b), (d) NOS in the hippocampus and cortex, respectively. These values are expressed as mean ± SD, *n* = 8; ^##^*P* < 0.01, compared with the normal control group; ^*∗*^*P* < 0.05 and ^*∗∗*^*P* < 0.01, compared with the model group.

As shown in Figure 7, the level of NO and the activity of NOS significantly increased in the hippocampus (*P* < 0.01) and cortex (*P* < 0.01) of rats in the model group. After the middle-dose and high-dose OMO treatment, the levels of NO decreased. Compared with the model group, the differences were significant (*P* < 0.05). However, there was no significant effect of OMO on NOS activity in the hippocampus and cortex (*P* > 0.01).

#### 3.2.5. Effects of OMO on Energy Metabolism Levels in the Hippocampus and Cerebral Cortex in AD Rats

**Figure 8:**
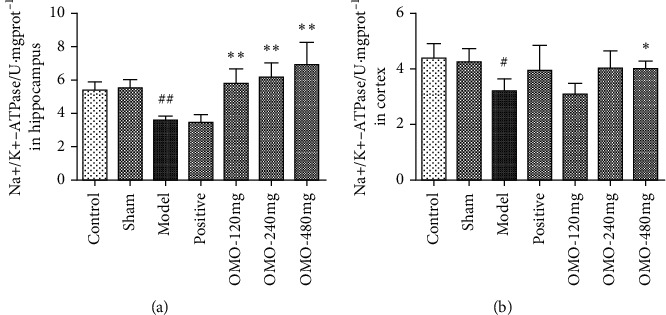
Effect of OMO on energy metabolism levels in the hippocampus and cerebral cortex of D-galactose and A*β*_25–35_-treated AD rats. The graph shows the level of Na+/K + -ATPase in the hippocampus (a) and cortex (b), respectively. These values are expressed as mean ± SD *n* = 8. ^#^*P* < 0.05 and ^##^*P* < 0.01, compared with the normal control group; ^*∗*^*P* < 0.05 and ^*∗∗*^*P* < 0.01, compared with the model group.

As shown in Figure 8, the activity of Na+/K + -ATPase in the hippocampus (*P* < 0.01) and cortex (*P* < 0.05) in the model group was significantly lower than that in the control group, suggesting that the d-galactose and A*β*_25–35_-induced AD model has a significant effect on the energy metabolism of neurons. The activity of Na+/K + -ATPase in the hippocampus significantly increased in all OMO groups (*P* < 0.01), while the activity of Na+/K + -ATPase in the cortex significantly increased in the high-dose OMO group (*P* < 0.05).

### 3.3. Effects of OMO on Monoamine Neurotransmitter Levels in the Hippocampus and Cerebral Cortex in AD Rats

#### 3.3.1. Effects of OMO on Monoamine Neurotransmitter Levels in the Hippocampus in AD Rats

**Figure 9:**
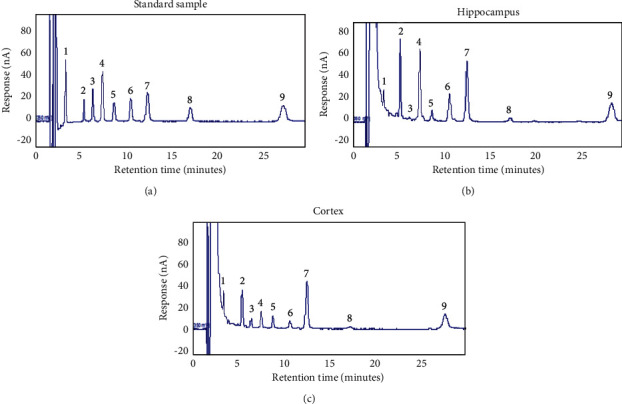
The neurotransmitters in the hippocampus and cortex were measured by HPLC-ECD. (a) Mixed standard; (b) hippocampus; (c) cortex. The ordinate represents the response intensity, and the abscissa represents the retention time. (1) L-DOPA; (2) NE; (3) E; (4) DOPAC; (5) DHBA (internal standard); (6) 5-HIAA; (7) DA; (8) HVA; (9) 5-HT.

**Figure 10:**
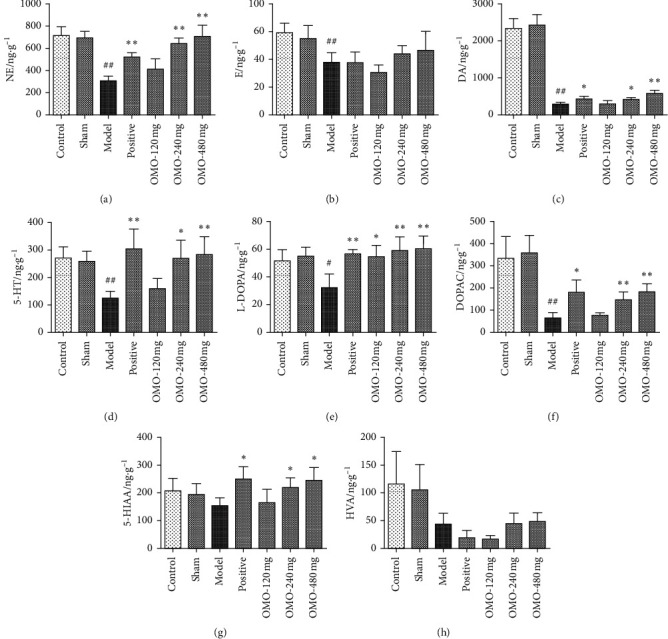
Effect of OMO on the monoamine neurotransmitter levels in the hippocampus of D-galactose and A*β*_25–35_-treated AD rats. The graphs show levels of (a) NE, (b) E, (c) DA, (d) 5-HT, (e) L-DOPA, (f) DOPAC, (g) 5-HIAA, and (h) HVA. These values are expressed as mean ± SD, *n* = 8. ^#^*P* < 0.05, ^##^*P* < 0.01, compared with the normal control group; ^*∗*^*P* < 0.05 and ^*∗∗*^*P* < 0.01 compared with the model group.

First, levodopa (L-DOPA), norepinephrine (NE), epinephrine (E), 3, 4-dihydroxyphenylacelic acid (DOPAC), dopamine (DA), 5-hydroxyindole-3-acetic acid (5-HIAA), homovanillic acid (HVA), and 5-hydroxytryptamine (5-HT) in the hippocampus and cortex were successfully measured by HPLC-ECD (Figure 9). Next, as shown in Figure 10 and compared with those in the normal control group, the levels of NE, E, DA, and 5-HT in the hippocampus in the model group significantly decreased (*P* < 0.01). After middle-dose and high-dose OMO treatment, the levels of NE (*P* < 0.01), DA (*P* < 0.05), and 5-HT (*P* < 0.05) significantly increased when compared with those in the model group. At the same time, the levels of L-DOPA (*P* < 0.05) and DOPAC (*P* < 0.01) in the hippocampus of the model group significantly decreased when compared with those in the normal control group. The levels of L-DOPA in the hippocampus significantly increased in all OMO groups (*P* < 0.05), while the levels of DOPAC (*P* < 0.05) and 5-HIAA (*P* < 0.01) in the hippocampus significantly increased in middle-dose and high-dose OMO groups. In the hippocampus, there was no significant difference in HVA between the normal control group and the model group and between the administration group and the model group.

#### 3.3.2. Effects of OMO on Monoamine Neurotransmitter Levels in the Cerebral Cortex in AD Rats

**Figure 11:**
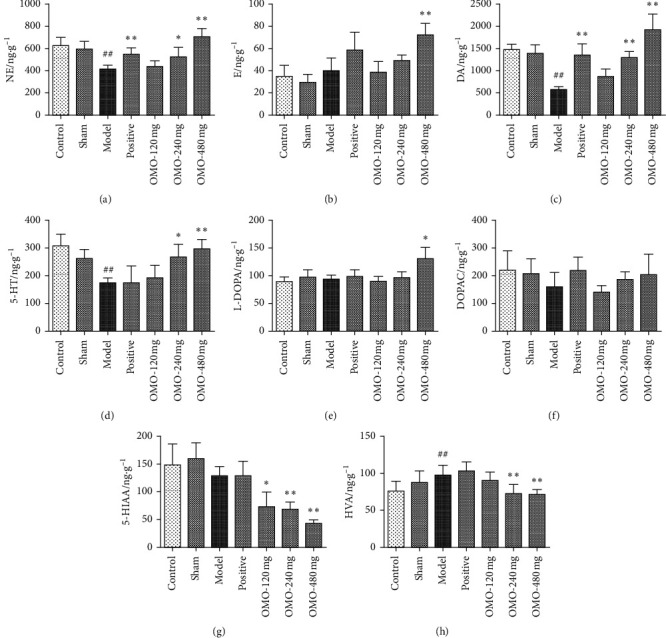
Effect of OMO on monoamine neurotransmitter levels in the cerebral cortex of D-galactose and A*β*_25–35_-treated AD rats. The graphs show the levels of (a) NE, (b) E, (c) DA, (d) 5-HT, (e) L-DOPA, (f) DOPAC, (g) 5-HIAA, and (h) HVA. These values are expressed as mean ± SD, *n* = 8. ^#^*P* < 0.05 and ^##^*P* < 0.01, compared with the normal control group; ^*∗*^*P* < 0.05 and ^*∗∗*^*P* < 0.01, compared with the model group.

As shown in Figure 11, the levels of NE, DA, and 5-HT in the cortex of the model group significantly decreased when compared to those in the normal control group (*P* < 0.01). After the middle-dose and high-dose OMO treatment, the levels of NE (*P* < 0.05), DA (*P* < 0.01), and 5-HT (*P* < 0.05) significantly increased, and the levels of E also significantly increased (*P* < 0.01) after the high-dose OMO treatment when compared to the levels in the model group. At the same time, compared with the normal control group, the model group exhibited a significant increase in levels of HVA (*P* < 0.01) in the cortex. The levels of L-DOPA in the cortex significantly increased in the high-dose OMO group (*P* < 0.05), and the levels of 5-HIAA significantly increased in all OMO groups (*P* < 0.05), while the levels of HVA significantly increased (*P* < 0.01) in middle-dose and high-dose OMO groups. In the hippocampus, there was no significant difference in DOPAC between the normal control group and the model group and between the administration group and the model group.

### 3.4. Effects of OMO on APP, Tau, SYP, and Caspase-3 Protein Levels in the Hippocampus of AD Rats

**Figure 12:**
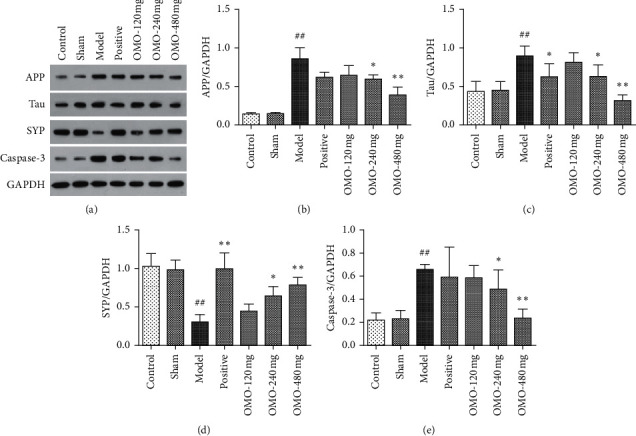
Effect of OMO on APP, tau, SYP, and caspase-3 protein levels in the hippocampus of D-galactose and A*β*_25–35_-treated AD rats. The Western blot analysis for APP, tau, SYP, and caspase-3 in the rat hippocampus after OMO treatment. The graphs show the levels of (b) APP, (c) tau, (d) SYP, and (e) caspase-3 when compared to the GAPDH levels. These values are expressed as mean ± SD, *n* = 6. ^##^*P* < 0.01, compared with the normal control group; ^*∗*^*P* < 0.05 and ^*∗∗*^*P* < 0.01, compared with the model group.

As shown in Figure 12, the expression of APP, tau, and caspase-3 protein in the hippocampus in the model group significantly increased (*P* < 0.01), and the SYP protein significantly decreased (*P* < 0.01), when compared to those in the normal control group. Furthermore, the expression levels of APP, tau, and caspase-3 protein decreased and the SYP protein increased in all OMO groups when compared to those in the model group. The differences were significant in the middle-dose (*P* < 0.05) and high-dose OMO groups (*P* < 0.01), suggesting that the OMO treatment can decrease the expression levels of APP, tau, and caspase-3 and increase the SYP protein in the hippocampus of AD rats, especially in the high-dose OMO group.

## 4. Discussion

AD is a complicated and serious neurodegenerative disease caused by various factors. Consequently, ideal AD animal models should not only simulate the behavioral changes observed in AD but also copy the changes in pathology, biochemistry, and neurotransmitters. Therefore, the investigators created an AD rat model via the combined administration of d-galactose and A*β*_25–35_. Specifically, long-term intraperitoneal injections of d-galactose accelerated the metabolism of rats, leading to metabolic disorders, and eventually resulting in aging [20]. After one injection of aggregating A*β*_25–35_ on both sides of the hippocampus, the rats exhibited disordered learning and memory after one week, establishing the AD animal model [21].

It has been demonstrated that oxidative stress is involved in many of the pathological processes of neurodegenerative diseases, including AD [22]. There was a definite correlation between AD and oxidative stress, and the degree of oxidative damage in brain tissue could be considered an early index of AD pathology. The SOD, CAT, GSH-Px, and MDA in the present study were recognized as oxidative stress markers closely related to AD [23]. Among these, SOD, CAT, and GSH-Px are the most important antioxidant enzymes in the human body, which can resist oxidation free radicals, regulate the metabolism of free radicals, and play a key role in the free radical scavenging system [24]. In addition, the content of MDA is also an important parameter that reflects the potential antioxidant capacity of the body, which can indirectly reflect the degree of tissue peroxidation damage [25]. The present data show that after the OMO treatment, the oxidative stress in the brain of animals in each dose group improved, suggesting that OMO could improve the oxidative stress level in the hippocampus and cortex of rats and protect brain tissue from oxidative damage. In addition, the activity of SOD in the treatment group was higher than that in the control group, which may be due to the fact that the activity of SOD in the brain of animals can be significantly increased by the long-term use of OMO extract. However, there was no significant difference in SOD activity between the two groups.

For patients with AD, the cholinergic neuron system has neurotransmitter deficits, and the cholinergic neurons were obviously damaged, especially in the cortex, hippocampus, nucleus basalis of Meynert, and septal area. The activity levels of choline acetyltransferase (CHAT) and AChE in the brain decreased, causing a decrease in Ach concentration. Numerous studies have shown that the cholinergic system is closely correlated to memory and learning ability [26]. Blocking the cholinergic system could cause memory and learning deficits similar to AD symptoms. Reinforcing the central cholinergic activity can improve the learning and memory abilities in aged individuals. Therefore, the change in the cholinergic system is closely correlated to the cognitive impairment observed in AD patients. According to the present results, medium-dose and high-dose OMO groups exhibited a reduced AChE activity in the hippocampus and cortex of AD model rats and an increase in Ach content, suggesting that OMO could protect the cholinergic system in AD model rats.

Most neurotransmitters in the central nervous system of mammals are amino acid neurotransmitters, including excitatory amino acids (EAAs, such as glutamate and aspartic acid) and inhibitory amino acids (IAAs, such as *γ*-GABA and glycine), which are important substances that regulate physiological activity. In recent years, the close relationship among EAAs, IAAs, AD morbidity, learning, and memory has drawn extensive attention. In general, it has been considered that EAAs can cause neurotoxicity. Furthermore, the release of large amounts of EAAs may lead to nerve cell damage and is correlated to the overloading of Ca^2+^ [27]. IAAs have a postsynaptic inhibitory function, which can protect nerves by reducing the internal flow of Ca^2+^. These present results show that OMO can lower the level of glutamate and increase the level of *γ*-GABA in the hippocampus and cortex of AD model rats. In addition, the medium-dose and high-dose groups achieved the best results, suggesting that OMO has a significant impact on amino acid neurotransmitter levels in the hippocampus and cortex of AD model rats.

The role of nitric oxide (NO) in AD has also drawn extensive attention. NO is a gas molecule with unstable properties that plays a role as a second messenger and neurotransmitter in the central nervous system and is involved in many physiological processes in the brain. However, NO can also cause neurotoxicity and nervous injury. NO is catalyzed and produced by NOS, and an increase in NO is closely correlated to learning and memory failure. In line with this effect, the retention defect is the main clinical feature of AD [28]. The present study revealed that NO and NOS decreased to a certain extent after OMO treatment. Furthermore, the medium-dose and high-dose groups had significantly lower levels of NO in the hippocampus and cortex in AD model rats.

Previous studies have already shown that glucose metabolism decreases in the temporal region of AD patients [29]. This change occurs in the early stage of AD. Furthermore, there are various types of energy metabolism defects in the brains of AD patients. In addition, the oxygenation efficiency of skin fibroblast glucose in AD patients decrease, and the activities of the pyruvate dehydrogenase complex and the *α*-oxoglutarate dehydrogenase complex decrease, activating the MAPK signaling pathway and causing mitochondrial metabolism disorder. According to the present experimental results, each dose significantly improved the hippocampal activity in AD model rats, while high-dose OMO improved the Na^+^/K^+^-ATPase activity in the cortex of AD model rats, suggesting that OMO can improve energy metabolism abnormalities in AD model rats.

Monoamine neurotransmitters include catecholamine compounds, indoleamine compounds, and their correlative metabolites (DA metabolic precursor L-DOPA, DA metabolites DOPAC and HVA, and 5-HT metabolite 5-HIAA). It has been shown that neurochemical changes in aging and AD are involved in several neurotransmitter systems, such as the adrenergic, dopaminergic, and 5-hydroxytryptamine systems [30, 31]. Under normal circumstances, the secretion of internal neurotransmitters remains at a certain level and in a harmonious proportion to maintain functional stability. With aging, the metabolism of monoamine neurotransmitters can become imbalanced. The levels of NE, DA, and 5-HT decrease, suggesting that the equilibrium between monoamine neurotransmitters in the brain is damaged during aging. Therefore, the investigators established a HPLC-ECD method for the separation and determination of these neurotransmitters and successfully completed the methodological validation [19]. The present data show that the levels of NE, E, DA, 5-HT, L-DOPA, and DOPAC in the hippocampus of model rats decreased, and the levels of NE, DA, and 5-HT decreased in the cortex, suggesting that the d-galactose and A*β*_25–35_-induced AD model leads to metabolic disorders related to monoamine neurotransmitters. After OMO treatment, the levels of NE, DA, 5-HT, L-DOPA, DOPAC, and 5-HIAA in the hippocampus of rats increased, while the levels of NE, DA, 5-HT, E (OMO high-dose group), and L-DOPA (OMO high-dose group) in the cortex of rats increased to different degrees, suggesting that OMO can regulate the metabolic levels of monoamine neurotransmitters in different brain areas of model animals in the present experiment. However, OMO had no effect on E and HVA in the hippocampus or DOPAC in the cortex. It was speculated that this is correlated to the related metabolic pathway: AD can disturb the metabolism of monoamine neurotransmitters in the brain, such as NE, DA, 5-HT, HVA, and 5-HIAA, especially the NE metabolism systems (NE/E) and the DA metabolism systems (L-DOPA/DA/DOPAC/HVA) [32–34]. Specifically, OMO has a significant impact on metabolic pathways of NE/E, L-DOPA/DA, 5-HT/5-HIAA, and DA/DOPAC, among others, in the rat hippocampus, and impacts metabolic pathways of L-DOPA/DA, 5-HT/5-HIAA, and DA/HVA, among others, in the cortex, suggesting that OMO can enhance learning and memory abilities by regulating the metabolic pathway of monoamine neurotransmitters in different brain areas.

The main pathological characteristics of AD are amyloid deposits in the brain, neurofibrillary tangles, vacuolar degeneration of neuronal granules, and neuronal loss. The main substance found in amyloid deposits and A*β* comes from the proteolysis of amyloid precursor protein (*β*-APP), which has a higher relative molecular mass. In addition to its normal physiological roles, APP and its metabolite also cause extensive neurotoxicity, which plays a key role in the pathogenesis of AD. Researchers have found that A*β* is generated after the degradation of APP, which in turn aggregates the outside of the cell to form senile plaques. These plaques subsequently lead to various cytotoxic effects, including changes in synaptic structure and function, the habituation of long-duration synaptic potentials, and neurodegeneration, by triggering various mechanisms of toxicity (activating caspases to trigger apoptosis, inflammatory reactions, oxidative stress, the axonal transport barrier, neurofibrillary tangles, and increased internal flow of Ca^2+^), eventually leading to apoptosis, autophagy, or necrocytosis. Abundant evidence indicates that proteins, such as APP, tau, SYP, and caspase-3, change during the pathogenic process of AD [35–38]. Based on Western blot detection, this research demonstrates that the long-term intraperitoneal injection of d-galactose and hippocampal injections of A*β*_25–35_ in both hemispheres would increase the APP, tau, and caspase-3 protein expression levels in the hippocampus of rats and inhibit the SYP protein expression. After the OMO treatment, the protein expression abnormalities were alleviated, and the high-dose OMO group achieved the most evident effect.

## 5. Conclusion

In the present study, the brain regions (hippocampus and cortex) in AD model animals were selected for the experimental study. The results revealed that OMO could significantly increase the learning and memory abilities of these rats by ameliorating oxidative stress and enhancing the energy metabolism and neurotransmitter levels. Thus, OMO has a neuroprotective effect on the hippocampus and cortex of AD model animals.

## Data Availability

The data used to support the findings of this study are included within the article.
